# Screen-Detected Breast Cancer Outcomes by Mammography Participation in Immediate Past Screening

**DOI:** 10.1001/jamanetworkopen.2025.35330

**Published:** 2025-10-03

**Authors:** Xinhe Mao, Wei He, Jose Tapia, Natalie Holowko, Jenny Bergqvist, Keith Humphreys, Kamila Czene

**Affiliations:** 1 Department of Medical Epidemiology and Biostatistics, Karolinska Institutet, Stockholm, Sweden; 2 Department of Nutrition and Food Hygiene, Children’s Hospital, Zhejiang University School of Medicine, National Clinical Research Center for Child Health, Hangzhou, China; 3 Chronic Disease Research Institute, School of Public Health, School of Medicine, Zhejiang University, Hangzhou, China; 4 The Ritchie Centre, Hudson Institute of Medical Research, Victoria, Australia; 5 Department of Oncology, Capio St Görans Hospital, Stockholm, Sweden

## Abstract

**Importance:**

Mammography screening is essential for the early detection of breast cancer; however, delayed detection among screen-detected breast cancers (SDBCs) is rarely studied.

**Objectives:**

To investigate whether women diagnosed with SDBC who missed the screening round immediately before the diagnostic round experience clinically significant delays in detection and whether tumor characteristics vary.

**Design, Setting, and Participants:**

This prospective register-based cohort study included all women diagnosed with SDBC in Stockholm, Sweden, between January 1, 1995, and February 28, 2020, with a follow-up until December 31, 2022. Data were analyzed from November 5, 2023, to May 27, 2024.

**Exposure:**

Nonparticipation in the screening immediately prior to the diagnostic round.

**Main Outcomes and Measures:**

Tumor characteristics and breast cancer–specific survival.

**Results:**

Among 8602 women with SDBC (median age at diagnosis, 61 [IQR, 55-66] years), 1482 (17.2%) did not attend the immediate past screening. Nonparticipants in the past screening were more likely to have larger tumors (adjusted odds ratio [AOR], 1.55 [95% CI, 1.37-1.76] for a tumor size ≥20 mm), lymph node involvement (AOR, 1.28 [95% CI, 1.12-1.45), and distant metastasis (AOR, 4.64 [95% CI, 2.10-10.29]) and less likely to have estrogen receptor–negative breast cancer (AOR, 0.74 [95% CI, 0.60-0.92]); however, there were no differences in progesterone receptor status (AOR, 0.96 [95% CI, 0.83-1.11]) or *ERBB2* (formerly *HER2* or *HER2/neu*) status (AOR, 1.00 [95% CI, 0.81-1.24]). In addition, these women experienced poorer breast cancer–specific survival, with an adjusted hazard ratio (AHR) of 1.33 (95% CI, 1.08-1.65). There was no association after adjusting for tumor characteristics (AHR, 1.11 [95% CI, 0.89-1.38]). Additionally, no association was found between nonparticipation in the second-to-last screening and tumor characteristics among those with screen-detected breast cancers (AHR, 0.98 [95% CI, 0.80-1.19] for stage II tumors or higher).

**Conclusions and Relevance:**

The findings of this cohort study suggest that some women with SDBC experience delayed detection and have clinically relevant worse outcomes. Future research is needed to investigate whether advancing the next mammography screening invitation date could enhance early detection and improve breast cancer outcomes in this population.

## Key Points

**Question:**

Is missing the preceding screening round associated with clinically significant detection delays among women with screen-detected breast cancer?

**Findings:**

In this cohort study of 8602 participants, women who did not participate in the immediate past screening round before the diagnostic round had worse tumor characteristics and consequently poorer breast cancer–specific survival during the next 20 years.

**Meaning:**

These findings suggest that women with screen-detected breast cancer who missed their immediate past screening experienced clinically relevant worse outcomes and that advancing the next invitation date for women who miss a screening may improve breast cancer outcomes in this population.

## Teaser

This cohort study investigates whether women diagnosed with screen-detected breast cancer who did not attend the screening round immediately before the screening that diagnosed their cancer have differences in tumor characteristics and survival compared with women who attended both screening rounds.

## Introduction

Mammography screening programs contribute to a reduced breast cancer mortality rate by detecting cancers at an early stage.^1,2,3^ Women diagnosed with breast cancer at a screening, commonly known as screen-detected breast cancer (SDBC), are generally considered to benefit most from screening. Compared with women with non-SDBC, women with SDBC are more likely to exhibit less aggressive tumor characteristics^4,5,6,7,8,9,10^ and, consequently, to have a better prognosis.^5,7,9,11^ Heterogeneity among women with SDBC has not been studied extensively, likely due to their generally favorable prognosis, although a considerable number of SDBC cases are diagnosed at stage III or IV.^6,12,13^ Therefore, it is crucial to identify which subgroups of women with SDBC experience late detection.

Only a small proportion of women attend every mammography screening invitation, with many attending irregularly.^14,15^ Consequently, some patients diagnosed with SDBC did not attend their immediate past screening. Therefore, it is reasonable to hypothesize that some women with SDBC might have already had detectable cancer by mammography at their immediate past screening visit. Had they attended that screening, these women might have been diagnosed with cancer at that time (ie, earlier).

Using data from the Breast Cancer Quality Register and the Stockholm Mammography Screening Program, we retrieved the full screening history of all women diagnosed with SDBC in Stockholm, Sweden. We investigated whether tumor characteristics differed depending on nonparticipation in the immediate past screening round prior to the diagnostic screening. We then further examined whether the prognosis of breast cancer among screen-detected cases differed based on nonparticipation in the immediate past screening. Additionally, using data from the general screening population in Stockholm, we tested our hypothesis that missing a screening round may delay identifying SDBC, thereby increasing cancer detection rates in the following screening round.

## Methods

### Data Source

In this cohort study, we used data from the Stockholm Mammography Screening Register and the Stockholm Breast Cancer Quality Register, which were linked using the unique Swedish Personal Identification Number. These were further linked with national registers including the Cause of Death Register, Migration Register, and the Longitudinal Integrated Database for Health Insurance and Labour Market Studies (LISA) database to obtain information on follow-up and sociodemographic characteristics. By leveraging these high-quality Swedish registers, we achieved virtually complete follow-up, with fewer than 5% missing data in most variables.^16,17,18,19^ A detailed description of the data sources and linkage process is provided in the eMethods in Supplement 1.

The regional ethical board of Karolinska Institutet approved this study. Swedish Ethical Review Authority waived the need for informed consent owing to the use of anonymized register data. The study adhered to the Strengthening the Reporting of Observational Studies in Epidemiology (STROBE) reporting guideline.

### Study Populations

#### Women With SDBC

SDBC cases in this study were defined as cancers detected following a mammography screening with a positive finding and before the next scheduled screening visit. We referred to the screening round in which breast cancer was diagnosed as the diagnostic round. We identified 11 286 women with invasive SDBC aged 40 to 76 years at the time of diagnosis between January 1, 1995, and February 28, 2020. After excluding women who had not received a screening invitation within the last 2 years (the usual screening interval) prior to the diagnostic screening, 8602 women remained in the final study population (eFigure in Supplement 1). This study population was drawn from women invited to mammography screening in Stockholm, where screening attendance has remained stable at approximately 72% throughout the study period, based on data from the present study.

#### General Screening Population

We assembled another study population to examine the cancer detection rate in 2 consecutive screening rounds in the general screening population. We identified 192 200 women who were invited for mammography screening in Stockholm in 2015 and retrieved follow-up data to determine the cancer detection rate for these women in both the 2015 screening and the subsequent screening round. The cancer detection rate was calculated as the number of cancers detected during a screening round (before the next scheduled screening) divided by the number of women screened. Given that the latest cancer diagnosis in our dataset was in early 2020, we chose 2015 for our invited screening because it was the most recent calendar year allowing for follow-up with 2 complete screening rounds (approximately 4 years). This study population was also drawn from the same screening program as the study population with SDBC.

### Exposure Definition

We retrieved data on participation at the mammography screening round immediately prior to the diagnostic screening, with our exposure defined as nonparticipation. In one analysis, we also assessed whether women participated in the second-to-last scheduled screening before the diagnostic round and determined screening patterns based on the immediate past 2 screening rounds.

### Outcome Definition

We retrieved data on tumor characteristics at diagnosis for each woman from the Breast Cancer Quality Register. Specifically, the following tumor characteristics were included: tumor size (<20 vs ≥20 mm), lymph node involvement, metastasis, TNM stage (I and II vs III, based on the Elston-Ellis grading system^20^), tumor grade, estrogen receptor (ER) status, progesterone receptor (PR) status, and *ERBB2* (formerly *HER2* or *HER2/neu*) status.

To investigate the prognosis of breast cancer, we sourced data on distant metastasis from the Breast Cancer Quality Register and death data from the Swedish Cause of Death register. Date on emigration was retrieved from the Migration Register. Women were followed up from their date of breast cancer diagnosis until distant metastasis, death, emigration, or December 31, 2022, whichever occurred first. Women who emigrated during follow-up were censored at the date of emigration. Breast cancer–specific survival was defined as the time from breast cancer diagnosis until death from breast cancer. Distant metastasis–free survival was defined as the time from breast cancer diagnosis until distant metastasis or death from breast cancer.

### Covariate Definition

We included demographic factors, socioeconomic status, and disease-related factors recorded closest to the invitation date of the immediate past screening invitation. Information on country of birth was retrieved from the Total Population Register. We retrieved data on highest educational level attained, individual income, marital status, and employment through the LISA database. Family history of breast cancer was defined as having a mother and/or sister who was diagnosed with the disease before the immediate past screening round prior to the diagnostic screening. Using data from the patient registers, we defined whether women had a comorbidity or obesity- or alcohol-related diseases. We calculated a weighted Charlson Comorbidity Index within 5 years of the immediate past screening round invitation, based on specific criteria.^21^ The list of obesity- or alcohol-related diseases accounted for in this study has been published previously.^22^

### Statistical Analysis

Data were analyzed from November 5, 2023, to May 27, 2024. We examined the association between nonparticipation in the immediate past round and tumor characteristics at cancer diagnosis using logistic or multinomial logistic regression models. We then used Kaplan-Meier estimation to examine breast cancer–specific and distant metastasis–free survival, comparing nonparticipants with participants. Furthermore, using Cox proportional hazards regression models, we investigated whether the association between such nonparticipation and breast cancer–related survival differed after minimal conditioning (age at diagnosis and calendar year), as well as conditioning for factors associated with nonparticipation and tumor characteristics. We also examined the association between nonparticipation patterns in the immediate past 2 rounds and tumor characteristics at cancer diagnosis using logistic regression. The proportional hazards assumption was assessed using the Schoenfeld residuals test, with no model violations observed.

Finally, using data from the general screening population, we calculated cancer detection rates among women who were invited to the screening in 2015 and in the subsequent round. We present these rates separately, per 1000 screenings.

All *P* values were 2 sided, and *P* < .05 was considered statistically significant. We conducted all analyses using SAS, version 9.4 (IBM Corporation), R, version 4.3.1 (R Program for Statistical Computing), and Stata, version 18.0 (StataCorp LLC).

## Results

### Women With SDBC

#### Baseline Characteristics

Among 8602 women with SDBC, the median age at breast cancer diagnosis was 61 (IQR, 55-66) years. Of these women, 1482 (17.2%) did not attend their immediate past scheduled screening round. Nonparticipants were more likely to be younger, unemployed, unmarried, and born outside Sweden and to have lower income and alcohol-related diseases (eTable 1 in Supplement 1).

#### Tumor Characteristics and Survival by Participation in the Immediate Past Screening Round

Among women with SDBC, we found that those who did not participate in the immediate past screening round were more likely to have a large tumor (adjusted odds ratio [AOR], 1.55 [95% CI, 1.37-1.76] for a tumor size ≥20 mm), lymph node involvement (AOR, 1.28 [95% CI, 1.12-1.45), metastasis (AOR, 4.64 [95% CI, 2.10-10.29]), and higher TNM stages at diagnosis (AORs, 1.63 [95% CI, 1.31-2.03] for stage III and 5.35 [95% CI, 2.41-11.86] for stage IV) and less likely to have ER-negative breast cancer (AOR, 0.74 [95% CI, 0.60-0.92]) compared with participants (Table 1). There was no difference in PR status (AOR, 0.96 [95% CI, 0.83-1.11]) or *ERBB2* status (AOR, 1.00 [95% CI, 0.81-1.24]) when comparing nonparticipants with participants (Table 1).

**Table 1.** Tumor Characteristics Among Women With Screen-Detected Breast Cancer

Characteristic	Immediate past screening, No. (%)^a^		AOR (95% CI)^b^
	Participants (n = 7120)	Nonparticipants (n = 1482)	
TNM stage			
I	4427 (63.5)	774 (53.9)	1 [Reference]
II	2126 (30.5)	523 (36.4)	1.33 (1.18-1.51)^c^
III	407 (5.8)	126 (8.8)	1.63 (1.31-2.03)^c^
IV	13 (0.2)	12 (0.8)	5.35 (2.41-11.86)^c^
Tumor size, mm			
<20	5312 (76.7)	949 (67.0)	1 [Reference]
≥20	1613 (23.3)	467 (33.0)	1.55 (1.37-1.76)^c^
Lymph node involvement			
No	5200 (76.3)	989 (70.2)	1 [Reference]
Yes	1617 (23.7)	420 (29.8)	1.28 (1.12-1.45)^c^
Distant metastasis			
No	6946 (99.8)	1436 (99.2)	1 [Reference]
Yes	13 (0.2)	12 (0.8)	4.64 (2.10-10.29)^c^
Tumor grade			
1 and 2	4328 (77.6)	896 (77.6)	1 [Reference]
3	1249 (22.4)	259 (22.4)	0.94 (0.80-1.09)
ER status			
Positive	5904 (90.2)	1272 (92.2)	1 [Reference]
Negative	644 (9.8)	107 (7.8)	0.74 (0.60-0.92)^c^
PR status			
Positive	5012 (76.8)	1070 (78.0)	1 [Reference]
Negative	1510 (23.2)	301 (22.0)	0.96 (0.83-1.11)
*ERBB2* status			
Negative	4411 (89.5)	919 (88.5)	1 [Reference]
Positive	517 (10.5)	120 (11.5)	1.00 (0.81-1.24)

Abbreviations: AOR, adjusted odds ratio; ER, estrogen receptor; PR, progesterone receptor.

^a^ Owing to missing data, the numbers in each category do not sum to the totals given in the column headings. Percentages have been rounded and therefore may not total 100.

^b^ Adjusted for age and calendar year of cancer diagnosis.

^c^ Significant at *P* < .05.

**Figure 1.**
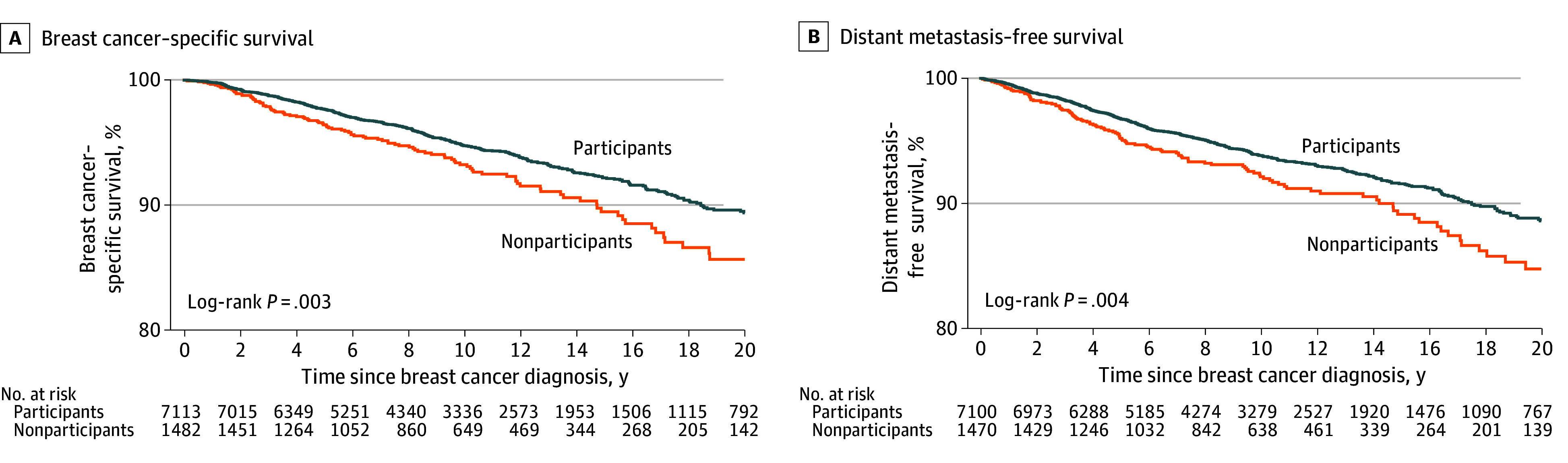
Breast Cancer Survival in Screen-Detected Cases by Participation in Immediate Past Screening Round Breast cancer–specific survival was defined as the time from breast cancer diagnosis until death from breast cancer. Distant metastasis–free survival was defined as the time from breast cancer diagnosis until distant metastasis or death from breast cancer. Analyses for estimating distant metastasis–free survival were conducted among patients with breast cancer without metastasis at the time of diagnosis.

The 20-year breast cancer–specific survival was 85.7% (95% CI, 82.2%-88.6%) among nonparticipants, which was significantly lower than the 89.4% (95% CI, 88.1%-90.6%) observed among participants in the immediate past screening round. Cox proportional hazards regression analyses (adjusted for age, calendar year of diagnosis, and all baseline factors associated with nonparticipation) showed that nonparticipants were more likely to have worse breast cancer–specific survival, with adjusted hazard ratios (AHRs) of 1.33 (95% CI, 1.08-1.65) (Table 2). This AHR decreased to 1.11 (95% CI, 0.89-1.38) after further adjusting for tumor characteristics, suggesting that the worse survival observed among nonparticipants was partially due to their worse tumor characteristics. Consistent findings were observed for distant metastasis–free survival (Figure 1 and Table 2).

**Table 2.** Breast Cancer Survival Among Women With Screen-Detected Cancer

Survival	No. of cases		AHR (95%CI)		
			Model		Further adjustment for tumor characteristics^c^
	Breast cancer	Event of interest	Base^a^	Full^b^	
**10-y Follow-up**					
Breast cancer specific					
Participants	7113	294	1 [Reference]	1 [Reference]	1 [Reference]
Nonparticipants	1482	80	1.38 (1.08-1.77)^d^	1.34 (1.04-1.72)^d^	1.11 (0.86-1.44)
Distant metastasis free^e^					
Participants	7100	358	1 [Reference]	1 [Reference]	1 [Reference]
Nonparticipants	1470	95	1.34 (1.07-1.68)^d^	1.34 (1.06-1.69)^d^	1.16 (0.92-1.47)
**Full follow-up**					
Breast cancer specific					
Participants	7113	420	1 [Reference]	1 [Reference]	1 [Reference]
Nonparticipants	1482	110	1.36 (1.10-1.68)^d^	1.33 (1.08-1.65)^d^	1.11 (0.89-1.38)
Distant metastasis free^d^					
Participants	7100	464	1 [Reference]	1 [Reference]	1 [Reference]
Nonparticipants	1470	120	1.32 (1.08-1.62)^d^	1.32 (1.07-1.62)^d^	1.14 (0.93-1.41)

Abbreviation: AHR, adjusted hazard ratio.

^a^ Adjusted for age at diagnosis and calendar year of diagnosis.

^b^ Additionally adjusted for baseline factors associated with nonparticipation, including disposable income, marital status, country of birth (Sweden or other), and alcohol-related diseases.

^c^ Additionally adjusted for tumor characteristics, including tumor size, lymph node involvement, metastasis, tumor grade, estrogen receptor status, progesterone receptor status, and *ERBB2* (formerly *HER2* or *HER2/neu*) status.

^d^ Significant at *P* < .05.

^e^ Analyses for estimating distant metastasis–free survival were conducted among patients with breast cancer without metastasis at the time of diagnosis.

#### Tumor Characteristics by Participation in the Immediate Past 2 Screening Rounds

Compared with women who attended the immediate past 2 screenings, those diagnosed with SDBC who did not participate the immediate past round (but participated the second-to-last round) had a higher TNM stage (AOR, 1.57 [95% CI, 1.32-1.88] for stage II tumors or higher) (Table 3). In contrast, no association was found between nonparticipation in the second-to-last screening and TNM stage (AOR, 0.98 [95% CI, 0.80-1.19] for tumors stage II or higher) (Table 3).

**Table 3.** Tumor Characteristics Among Women With Screen-Detected Breast Cancer^a^

Mammography screening participation		No. of breast cancer cases (n = 6726)	TNM stage, No. (%)		AOR (95%CI)^b^
Second-to-last round	Immediate past round		I	II-IV	
Yes	Yes	5150	3291 (63.9)	1859 (36.1)	1 [Reference]
Yes	No	550	284 (51.6)	266 (48.4)	1.57 (1.32-1.88)^c^
No	Yes	480	304 (63.3)	176 (36.7)	0.98 (0.80-1.19)
No	No	546	293 (53.7)	253 (46.3)	1.45 (1.21-1.73)^c^

Abbreviation: AOR, adjusted odds ratio.

^a^ The table structure of 5 columns reading down without rows reading across emphasizes the timing of screening participation (second-to-last round vs immediate past round) rather than the participation patterns.

^b^ Adjusted for age and calendar year of cancer diagnosis.

^c^ Significant at *P* < .05.

### General Screening Population

#### Cancer Detection Rates

**Figure 2.**
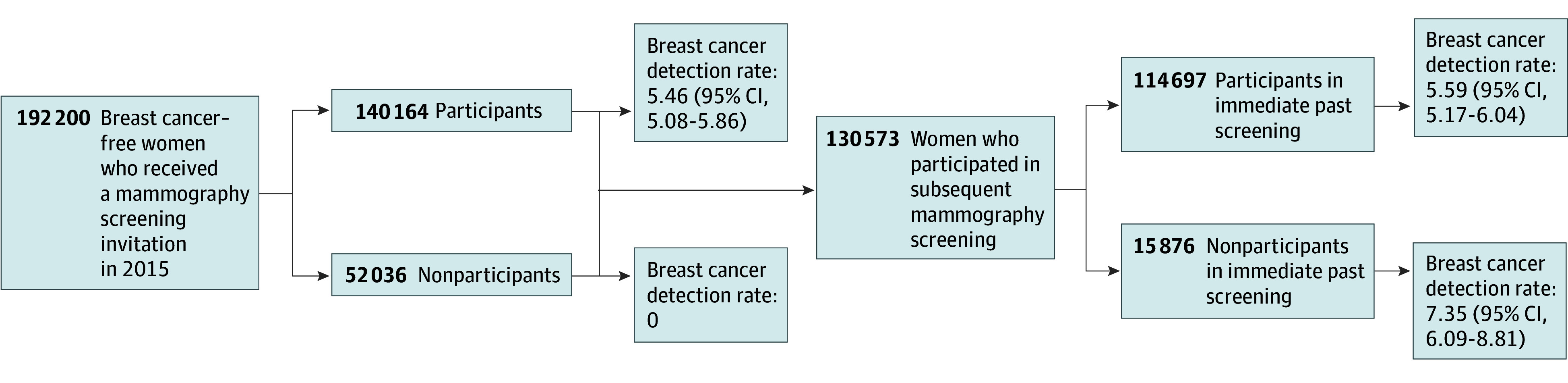
Breast Cancer Detection Rate Per 1000 in 2 Consecutive Rounds in General Screening Population The year 2015 was chosen because it was the most recent calendar year that permitted 2 complete screening rounds (about 4 years) during the follow-up period for cancer diagnosis (which ended in early 2020). Parenthetical data after detection rates are shown as 95% CIs.

Figure 2 shows the cancer detection rates during 2 consecutive screening rounds among the general screening population: the 2015 round and the subsequent round. In the 2015 round, the cancer detection rate was 5.46 (95% CI, 5.08-5.86) per 1000 screenings for women who participated and 0 per 1000 screenings for nonparticipants. In the following screening round, the detection rate among women who participated in 2015 was 5.59 (95% CI, 5.17-6.04) per 1000 screenings, which was significantly lower than the 7.35 (95% CI, 6.09-8.81) per 1000 screenings for those who did not participate in 2015 (*P* = .02).This finding suggests that missing a screening could delay cancer detection, leading to higher cancer detection rates in the following screening rounds.

When stratifying by age at screening, the differences between participants and nonparticipants were more pronounced in women 55 years and older compared with younger women (eTable 2 in Supplement 1). In addition, when examining the ER-specific cancer detection rate in following screening round, we observed notable differences in the detection rate of ER-positive cancer between 2015 participants and nonparticipants (4.36 [95% CI, 3.99-4.76] and 6.10 [95% CI, 4.95-7.43] per 1000 screenings, respectively; *P* = .006). In contrast, no significant difference in detection rate of ER-negative cancer was observed (0.38 [95% CI, 0.28-0.51] and 0.50 [95% CI, 0.22-0.99] per 1000 screenings, respectively; *P* = .59).

## Discussion

In this large population-based cohort study, we found that even among women who were diagnosed with breast cancer during screening, nearly 20% of these patients did not attend in the immediate past screening occasion before the diagnostic round. Compared with screening participants, these nonparticipants were more likely to have aggressive tumor characteristics and poorer breast cancer–specific survival. These findings suggest that for these women, having missed the most recent screening before the diagnostic round could have led to a meaningful delayed detection of breast cancer. Supporting this, a separate analysis of the general screening population indicated that women who missed one screening round had a significant higher rate of cancer detection in the subsequent round than those who participated in both screening rounds.

To our knowledge, this study is the first to investigate tumor heterogeneity and survival among women with SDBC while considering their immediate past screening attendance. Previous research has also highlighted the impact of screening interval length on breast cancer detection and outcomes. For example, shorter screening intervals have been associated with smaller tumors and earlier-stage diagnoses,^23,24^ which supports the biological plausibility of our findings.

Our novel results from following aspects supported our hypothesis that even among women with SDBC, nonparticipation in immediate past screening delays cancer detection. First, in line with previous results, we identified several factors associated with nonattendance, including lower socioeconomic status, immigrant background, and alcohol-related diseases.^15,25,26^ However, our study extended beyond previous research by examining tumor characteristics in relation to detailed screening history. We found that compared with women who participated in the immediate past screening round, nonparticipants were more likely to have a larger tumor size, lymph node involvement, metastasis, and a higher TNM cancer stage, indicating longer tumor development. In addition, we found that tumor characteristics differed based on nonparticipation in the immediate screening before the diagnostic round, but not based on nonparticipation in the second-to-last screening round. This result directly supports the hypothesis that differences in tumor characteristics observed between the 2 groups were mainly due to tumor progression, rather than general behavioral characteristics associated with screening nonparticipation. These results underscore the potential value of targeted interventions for high-risk groups, such as offering shorter reinvitation intervals or additional outreach following a missed screening appointment.

Second, we found that among women with SDBC, those who did not participate in the immediate past screening had worse survival than those who did; however, the survival differences diminished after adjusting for tumor characteristics. Thus, the observed survival differences were likely due to nonparticipants having more advanced tumor characteristics. These findings highlight the clinical significance of delayed detection among this group of patients with breast cancer, which is affecting survival. Because our study is the first, to our knowledge, to examine the association between nonparticipation in the most recent screening round and survival in SDBC, no previous studies are directly comparable. One earlier study in the broader screening context reported that women who attended 2 consecutive mammography rounds prior to diagnosis had significantly lower breast cancer mortality than those who attended only one.^27^ However, that study analyzed both SDBC and symptomatic cases together, and data on mode of detection were not available.

Third, previous studies have found that ER-positive breast cancer, which is generally slow growing, is more likely to be detected during screening.^28,29,30^ Consequently, nonparticipation in prior screening rounds may lead to a delay in detecting ER-positive breast cancer. Consistent with this notion, our investigation revealed a higher proportion of ER-positive cancers among women who did not participate in the immediate past screening round compared with those who did. Our results imply that attending the previous screening might have facilitated earlier detection of these cancers.

Fourth, on examining cancer detection rates in the general screening population (based on participation in 1 round), we found that nonparticipants had a higher cancer detection rate in the subsequent round compared with participants. Specifically, elevated cancer detection rates were primarily due to higher detection rates of ER-positive cancers. This aligns with our findings from patients with breast cancer and directly supports our hypothesis that nonparticipation in screening may be associated with delayed detection.

Our study was based on data from Stockholm, Sweden. Because screening attendance rates can be higher or lower in rural compared with urban areas, depending on the country,^31,32,33,34^ our results should be interpreted within the context of national and rural-urban differences. Notably, in two-thirds of European countries, mammography screening participation rates fell below 70%, which is lower than that in Sweden.^35^ Consequently, the proportion of patients with SDBC who did not attend their last invited screening round is assumed to be even higher in other parts of Europe, indicating a potentially more serious issue outside Sweden.

### Strengths and Limitations

This study’s strengths enabled us to address the research question comprehensively. By using complete screening data from the Stockholm Mammography Screening Register, we were uniquely positioned to accurately identify cases of SDBC along with their screening history and outcomes, supported by a substantial sample size. Additionally, access to multiple Swedish national registers, including the Swedish Cause of Death Register and the Stockholm Breast Cancer Quality Register, provided us with detailed tumor characteristics and virtually complete follow-up data for 20 years, enabling a thorough investigation of survival differences. The integration of various population-based registers also enabled us to account for a wide range of sociodemographic factors and patient characteristics into our analyses.

There are also noteworthy limitations of the study. Variations in screening practices and attendance across different countries may limit the generalizability of our findings. Another possible concern is lead-time bias. Lead-time bias is an important issue that should be addressed when comparing the survival of SDBC with that of symptomatic cancers.^36,37^ However, given that all women had invasive SDBC, the effect of lead-time bias is expected to be less substantial than in studies comparing SDBC with symptom-detected cancers. We also hypothesize that lead-time bias may bias our survival time comparisons toward the null. This is because SDBC in women who missed immediate past screening are likely to include more slow-growing tumors, compared with those in women who regularly attend screenings.

## Conclusions

In this cohort study of patients with SDBC, we found that nonattendance at the immediate past screening round was associated with more advanced tumors and poorer survival. These findings highlight the importance of regular participation. Further research is needed to determine whether sending the next screening invitation at shorter intervals to those who missed their last screening can facilitate earlier cancer detection.

## Notes

Supplement 1. **eFigure.** Flowchart of Study Participants **eTable 1.** Baseline Characteristics of Women With Screen-Detected Breast Cancer **eTable 2.** Detection Rate of Breast Cancer (per 1000 Screenings) in 2 Consecutive Screening Rounds in the General Screening Population **eMethods.** Data Sources
eReferences

Supplement 2.
Data Sharing Statement
