# Supplementary material for: Screen-Detected Breast Cancer Outcomes by Mammography Participation in Immediate Past Screening
Source: JAMA Netw Open. 2025 Oct 3;8(10):e2535330. doi: 10.1001/jamanetworkopen.2025.35330 (PMC12495498; doi:10.1001/jamanetworkopen.2025.35330)
Supplement: Supplement 1. — eFigure. Flowchart of Study Participants eTable 1. Baseline Characteristics of Women With Screen-Detected Breast Cancer eTable 2. Detection Rate of Breast Cancer (per 1000 Screenings) in 2 Consecutive Screening Rounds in the General Screening Population eMethods. Data Sources eReferences [file jamanetwopen-e2535330-s001.pdf]

Supplementary Online Content

Mao X, He W, Tapia J, et al. Screen-detected breast cancer outcomes by mammography participation in immediate past screening. *JAMA Netw Open.* 2025;8(X):e2535330. doi:10.1001/jamanetworkopen.2025.35330

eFigure. Flowchart of Study Participants
eTable 1. Baseline Characteristics of Women With Screen-Detected Breast Cancer
eTable 2. Detection Rate of Breast Cancer (per 1000 Screenings) in 2 Consecutive Screening Rounds in the General Screening Population
eMethods. Data Sources
eReferences

This supplementary material has been provided by the authors to give readers additional information about their work.

© 2025 Mao X et al. *JAMA Network Open*.

eFigure. Flowchart of Study Participants

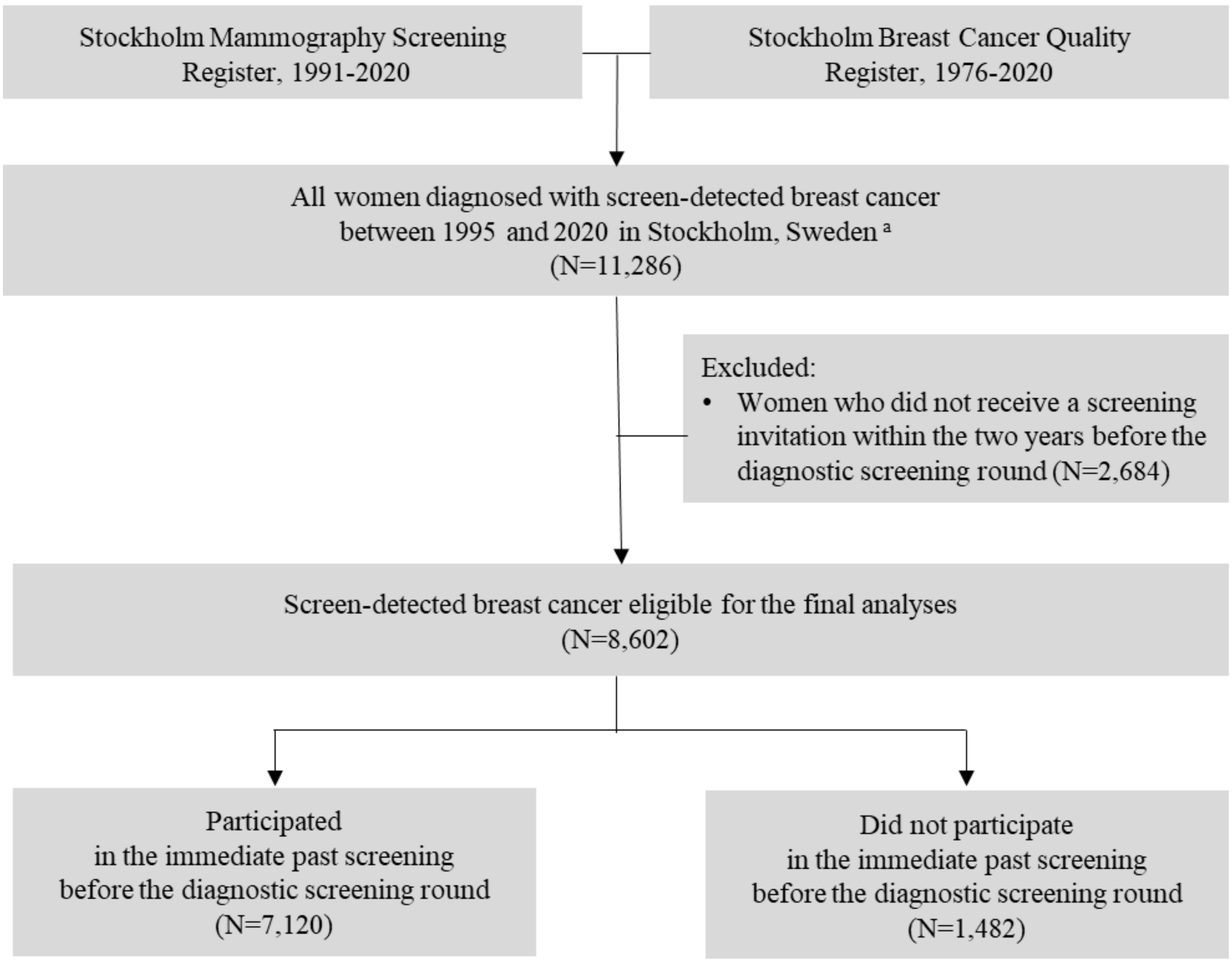

Note: The study population was followed up until December 31st, 2022.
Women did not receive a screening invitation within two years prior to their diagnostic round due to: (1) having recently moved to Stockholm and not yet entered the regional screening cycle; (2) being aged 40 or 50 and receiving their first invitation; (3) being aged 41–50 during 2005–2009, when this group was gradually added to the program in Stockholm; or (4) being over age 71 shortly after the 2012 expansion of eligibility

© 2025 Mao X et al. *JAMA Network Open*.

eTable 1. Baseline Characteristics of Women With Screen-Detected Breast Cancer

Non-participants vs. participants in the immediate past screening round before the diagnostic screening round.

	Immediate past screening		P-value [a]
	Participants (n=7,120; %)	Non-participants (n=1,482; %)	
Age at breast cancer diagnosis (years)			
40-49	644 (9.0)	215 (14.5)	<.001
50-59	2,149 (30.2)	560 (37.8)	
60-76	4,327 (60.8)	707 (47.7)	
Calendar year of last mammography screening			
1995-2003	1,417 (19.9)	280 (18.9)	0.656
2004-2012	2,384 (33.5)	498 (33.6)	
2013-2020	3,319 (46.6)	704 (47.5)	
**Common factors** [b]			
Education (years)			
⩽9	1,199 (16.9)	277 (18.9)	0.124
10-12	2,909 (41.0)	602 (41.1)	
>12	2,985 (42.1)	585 (40.0)	
Disposable income, own			
0-19.9%	1,009 (15.4)	308 (22.7)	<.001
20-39.9%	1,251 (19.1)	287 (21.2)	
40-59.9%	1,417 (21.6)	253 (18.7)	
60-79.9%	1,464 (22.3)	266 (19.6)	
80-100%	1,420 (21.6)	242 (17.8)	
Employment, binary			
Unemployed	2,601 (36.5)	561 (37.9)	0.336
Employed	4,519 (63.5)	921 (62.1)	
Marital status			
Not married	1,143 (16.1)	346 (23.3)	<.001
Married/ partnered	4,062 (57.1)	659 (44.5)	
Divorced	1,487 (20.9)	392 (26.5)	
Widowed/surviving partner	428 (6.0)	85 (5.7)	
Born in Sweden			
No	1,336 (18.8)	416 (28.1)	<.001
Yes	5,784 (81.2)	1,066 (71.9)	
Family history of breast cancer [c]			
No	6,326 (88.8)	1,332 (89.9)	0.248
Yes	794 (11.2)	150 (10.1)	
**Disease related factors** [d]			
Charlson comorbidity index			
0	6,461 (90.7)	1,341 (90.5)	0.384
1-2	607 (8.5)	125 (8.4)	
>2	52 (0.7)	16 (1.1)	
Obesity related diseases			
No	6,120 (86.0)	1,275 (86.0)	0.938

© 2025 Mao X et al. *JAMA Network Open*.

	Immediate past screening		P-value [a]
	Participants (n=7,120; %)	Non-participants (n=1,482; %)	
Yes	1,000 (14.0)	207 (14.0)	
Alcohol related diseases			
No	6,873 (96.5)	1,356 (91.5)	<.001
Yes	247 (3.5)	126 (8.5)	

Note: [a] P-values from chi-squared tests.
[b] Information closest to the immediate past screening invitation was used for each common factor.
[c] Among women who were born in Sweden, family history of breast cancer was defined as any mothers or full-sibling sisters of women who had a record of breast cancer in the Swedish Cancer Register prior to the women's immediate past screening invitation.
[d] Disease-related variables were assessed before the immediate past screening invitation for each woman. The weighted Charlson comorbidity index within the five years before the immediate past screening was calculated and used.

© 2025 Mao X et al. *JAMA Network Open*.

eTable 2. Detection Rate of Breast Cancer (per 1000 Screenings) in 2 Consecutive Screening Rounds in the General Screening Population

Non-participants vs. participants in the first of the two screening rounds, by age of women at the first screening.

	Detection rate: per 1000 screenings (95%CI)	
	Screening in 2015	The subsequent round
**Women aged <55 years at mammogram**		
Participants in 2015	2.68 (2.28 to 3.12)	3.04 (2.58 to 3.56)
Non- participants in 2015	0.00 (. to .)	3.91 (2.74 to 5.40)
**Women aged >=55 years at mammogram**		
Participants in 2015	7.64 (7.05 to 8.28)	7.60 (6.95 to 8.30)
Non- participants in 2015	0.00 (. to .)	12.10 (9.62 to 15.02)

Note: Calendar year 2015 was chosen as it was the most recent calendar year that allowed us to follow two complete screening rounds (about 4 years), as the follow-up for cancer diagnosis in our dataset was to early 2020.

© 2025 Mao X et al. *JAMA Network Open*.

eMethods. Data Sources

The Stockholm Mammography Screening Register contains records of all screening invitations, attendances, and results in the Stockholm region since 1989, including the exact dates of invitations and participation. Women aged 50–69 years were invited every 24 months from 1989 onward, with the age range extended to 40–74 years over time. Women who miss a round do not receive a reminder but are invited again after the regular interval [1,2].

The Breast Cancer Quality Register records all breast cancer diagnoses since 1976 in Stockholm, with detailed tumor characteristics, date of diagnosis, treatment, recurrence, and survival information [3,4].

These registers were linked through the unique Swedish Personal Identification Number, and further linked with:

- The Cause of Death Register. It has recorded virtually all deaths since 1952, including the date and cause of death for each individual [5]. This was used for mortality data.
- The Total Population Register. Initiated in 1967, it records each individual's country of birth, as well as immigration and emigration data—this migration data is referred to as the Migration Register [6]. It was used to obtain migration data and country of birth.
- The Multi-Generation Register. It includes all individuals born after 1932 who were still alive in 1961. Parental information was recorded, allowing siblings to be linked. In this register, 3% of individuals are missing information on their mothers and 5% are missing information on their fathers [7]. It was used to define family history of breast cancer.
- The National Patient Register. It provides hospital and outpatient diagnoses, with complete coverage of inpatient records since 1987 and of outpatient records since 2001 [8]. It was used to measure comorbidities and to identify obesity and alcohol-related diseases.
- The Longitudinal Integrated Database for Health Insurance and Labour Market Studies (LISA) contains information on socioeconomic position [9]. It was used to obtain data on income, employment, education, and marital status.

eReferences

1. Lind H, Svane G, Kemetli L, Törnberg S. Breast Cancer Screening Program in Stockholm County, Sweden - Aspects of Organization and Quality Assurance. *Breast care (Basel, Switzerland)*. 2010;5(5):353-357.
2. Lidbrink EK, Törnberg SA, Azavedo EM, et al. The general mammography screening program in Stockholm. Organisation and first-round results. *Acta oncologica (Stockholm, Sweden)*. 1994;33(4):353-8. doi:10.3109/02841869409098428
3. MATTSSON B, RUTQVIST LE, WALLGREN A. Undernotification of diagnosed cancer cases to the Stockholm Cancer Registry. *International journal of epidemiology*. 1985;14(1):64-69.
4. Löfgren L, Eloranta S, Krawiec K, et al. Validation of data quality in the Swedish National Register for Breast Cancer. *BMC public health*. May 2 2019;19(1):495. doi:10.1186/s12889-019-6846-6
5. Brooke HL, Talback M, Hornblad J, et al. The Swedish cause of death register. *European journal of epidemiology*. Sep 2017;32(9):765-773. doi:10.1007/s10654-017-0316-1
6. Ludvigsson JF, Almqvist C, Bonamy AK, et al. Registers of the Swedish total population and their use in medical research. *European journal of epidemiology*. Feb 2016;31(2):125-36. doi:10.1007/s10654-016-0117-y
7. Ekbom A. The Swedish Multi-generation Register. *Methods in molecular biology (Clifton, NJ)*. 2011;675:215-20. doi:10.1007/978-1-59745-423-0_10
8. Ludvigsson JF, Andersson E, Ekbom A, et al. External review and validation of the Swedish national inpatient register. *BMC public health*. 2011/06/09 2011;11(1):450. doi:10.1186/1471-2458-11-450
9. Ludvigsson JF, Svedberg P, Olén O, Bruze G, Neovius M. The longitudinal integrated database for health insurance and labour market studies (LISA) and its use in medical research. *European journal of epidemiology*. Apr 2019;34(4):423-437. doi:10.1007/s10654-019-00511-8
