# Supplementary material for: Screen-Detected Breast Cancer Outcomes by Mammography Participation in Immediate Past Screening
Source: JAMA Netw Open. 2025 Oct 3;8(10):e2535330. doi: 10.1001/jamanetworkopen.2025.35330 (PMC12495498; doi:10.1001/jamanetworkopen.2025.35330)
Supplement: Supplement 2. — Data Sharing Statement [file jamanetwopen-e2535330-s002.pdf]

Data Sharing Statement

Mao. Screen-Detected Breast Cancer Outcomes by Mammography Participation in Immediate Past Screening. *JAMA Netw Open*. Published October 03, 2025.
doi:10.1001/jamanetworkopen.2025.35330

Data
Data available: No

Additional Information
Explanation for why data not available: Data are not possible to share due to GDPR within the EU.
